# Association Between Cellular Hydration Patterns and Hydroelectrolytic Regulation with Muscle Strength in Older Adults

**DOI:** 10.3390/nu18050850

**Published:** 2026-03-05

**Authors:** Isabel Lorenzo, Mateu Serra-Prat, Esther Mur-Gimeno, Lluis Guirao, Juan Carlos Yébenes

**Affiliations:** 1 Consorci Sanitari del Maresme, 08304 Mataró, Spain; mserra@csdm.cat (M.S.-P.); jyebenes@csdm.cat (J.C.Y.); 2 Doctoral School, Universitat de Vic-Central de Catalunya, 08500 Vic, Spain; 3 Centro de Investigación Biomédica en Red de Enfermedades Hepáticas y Digestivas (CIBERehd), Instituto de Salud Carlos III, 08036 Barcelona, Spain; 4 VITAE Escola Universitària de l’Esport, Universitat Abat Oliba (CEU), Balmes 209, 08006 Barcelona, Spain; esther.mur@escolavitae.com; 5 Hospital Universitari Mutua de Terrassa, 08221 Terrasa, Spain; llguirao@gmail.com

**Keywords:** muscle health, cellular hydration, nutrition, electrolyte regulation, functional performance

## Abstract

**Introduction**: Muscle function is influenced by hydroelectrolytic mechanisms that regulate cellular volume beyond isolated plasma electrolyte concentrations. However, the role of integrated hydration and electrolyte regulation profiles in muscle function among older adults remains insufficiently understood. **Objective**: To identify which physiological domains of hydroelectrolytic regulation are most strongly associated with muscle strength and functional performance in community-dwelling older adults. **Methods**: A cross-sectional study was conducted in 96 community-dwelling individuals aged ≥ 70 years. Markers of cellular hydration and membrane integrity were assessed using bioelectrical impedance analysis, together with first-morning fasting plasma and urinary sodium and chloride concentrations. Principal component analysis (PCA) was applied as a data-driven approach to identify latent domains of coordinated hydroelectrolytic regulation. Associations between component scores and handgrip strength and Timed Up and Go (TUG) were examined using two sequential multivariable regression models: Model 1 adjusted for sex and fat-free mass index (FFMI); Model 2 additionally adjusted for age, hypertension, and diuretic use. **Results**: Three principal components were retained, explaining 77.5% of total variance: PC1 (renal–cellular domain), PC2 (plasma electrolyte domain), and PC3 (cellular volume domain). For handgrip strength, Model 1 showed significant associations for PC3 (β = 0.152; *p* = 0.025) and PC1 (β = 0.180; *p* = 0.050). In Model 2, only PC3 remained independently associated (β = 0.146; *p* = 0.036). For TUG, Model 1 showed associations for PC1 (β = −0.262; *p* = 0.049) and PC3 (β = −0.238; *p* = 0.015). In Model 2, PC1 (β = −0.308; *p* = 0.019) and PC2 (β = −0.190; *p* = 0.046) remained independently associated, whereas PC3 was not. **Conclusions**: Maximal force production appears primarily associated with cellular volume regulation, whereas functional performance reflects broader multi-compartmental hydroelectrolytic integration involving renal and plasma domains. These findings suggest that multidimensional hydration profiling may complement isolated biochemical markers in the functional assessment of older adults, warranting validation in longitudinal studies.

## 1. Introduction

Skeletal muscle function arises from the integration of multiple physiological layers, ranging from neuromuscular excitability to energy metabolism and tissue architecture [1,2,3]. Although classical determinants, such as nutrition, physical activity and metabolic or cardiovascular comorbidities, account for part of the interindividual variability, an additional and comparatively understudied dimension remains: the cellular microenvironment that sustains contractility and metabolic efficiency [4,5,6]. In particular, cellular and extracellular hydration status, together with osmotic balance, influence fundamental processes including enzyme kinetics, cytoskeletal organization and excitation–contraction coupling [7,8]. Experimental evidence further indicates that sustained increases in water intake may induce measurable changes in systemic metabolomic profiles, suggesting that hydration status can modulate metabolic pathways beyond its role in osmotic equilibrium [9]. Accordingly, disturbances in water and electrolyte regulation may be associated with changes in muscle function [8]. This issue is particularly relevant in older adults, in whom ageing is associated with impaired thirst perception, reduced renal concentrating capacity, and increased exposure to comorbidities and pharmacological treatments that may alter water and electrolyte homeostasis [10]. From this perspective, hydration should not be interpreted merely as total body water content, but as the integration of biochemical markers and bioelectrical parameters. Bioelectrical impedance-derived measures do not directly quantify cellular volume; rather, they provide indirect information on tissue electrical properties, influenced by body composition, fluid distribution, and membrane characteristics. When interpreted within this physiological framework, these signals may provide insight into cellular volume homeostasis [11].

The maintenance of cellular volume is essential for preserving myocyte structure, metabolism and contractile capacity [11,12]. Body water regulation does not operate as a single, uniform system, but instead functions across different levels. At the systemic level, osmolality is regulated by hypothalamic osmoreceptors and the vasopressin–kidney axis, which coordinate thirst and renal water reabsorption. This regulation is reflected predominantly in the plasma compartment [13,14]. In contrast, cells possess rapid and locally regulated mechanisms of volume control, largely mediated by transmembrane sodium and chloride fluxes, which enable the preservation of cellular structure and function [15,16]. The compartmental nature of this regulation implies that systemic markers may not always fully reflect compartment-specific alterations in cellular hydration [17]. Whereas osmolality reflects global osmotic balance, electrolyte concentration patterns across plasma and urine may capture complementary aspects of compartmental regulation [18]. In older adults, mild or chronic dehydration has been shown to be difficult to detect using isolated clinical signs or conventional biochemical parameters, given their limited diagnostic sensitivity in this population [19,20].

From a physiological standpoint, hydration status can therefore be understood as the integration of several complementary domains: (i) cellular hydration and integrity, reflecting the balance between the intracellular and extracellular compartments; (ii) the systemic osmotic environment, which determines plasma tonicity; and (iii) renal concentrating and excretory capacity, which dynamically modulates water and electrolyte availability. In the present study, principal component analysis (PCA) was applied as a data-driven approach to empirically approximate these interrelated physiological domains within a multivariate framework, allowing identification of coordinated variability patterns without imposing predefined assumptions about compartmental structure.

With ageing, and in the presence of chronic comorbidities such as hypertension, type 2 diabetes, mild renal impairment and prolonged use of certain medications, including diuretics and antidiabetics, this balance may become disrupted. This could contribute to a state of low-grade or subclinical dehydration, characterized by subtle shifts in intracellular water distribution and adaptive electrolyte responses aimed at maintaining osmotic equilibrium, which have been associated with alterations in muscle metabolism and functional capacity [21,22,23,24,25,26,27,28].

In clinical practice, hydration status is commonly evaluated using plasma-based parameters, such as sodium concentration or osmolality, which primarily reflect systemic water balance, while urinary or clinical indices are incorporated in selected contexts (17). However, this approach provides only a partial view, as it does not inform on the distribution of water across body compartments or on the state of cellular hydration. In this context, combining plasma and urinary markers with bioelectrical indicators allows a more comprehensive assessment of water regulation by integrating information on renal, plasma and cellular responses.

The aim of this study was to identify hydroelectrolytic profiles using multivariate analysis and to examine their associations with muscle strength and functional performance in community-dwelling older adults. A secondary aim was to evaluate whether these associations remained consistent after adjustment for key clinical covariates related to fluid and electrolyte regulation.

## 2. Materials and Methods

### 2.1. Study Design and Participants

This cross-sectional observational study was conducted within the framework of the AQUAM project and included 96 community-dwelling individuals aged ≥ 70 years. Participants were recruited from primary care registries within the catchment area of the Consorci Sanitari del Maresme (CSdM), Catalonia, Spain. Potentially eligible individuals were identified from primary care patient lists and contacted by telephone. Recruitment followed a non-probabilistic convenience sampling approach based on voluntary participation. Although non-probabilistic, recruitment was based on systematic identification from primary care registries.

Individuals who expressed interest attended an in-person screening visit at their primary care center, where eligibility was confirmed and written informed consent was obtained. Inclusion criteria were age ≥ 70 years, community-dwelling status, independent ambulation, and provision of informed consent. Exclusion criteria were active malignancy or palliative care; life expectancy < 6 months; diagnosed muscle diseases (e.g., muscular dystrophies or motor neurological deficits); bilateral hip or knee prostheses; presence of cardiac pacemakers or implantable devices; and severe chronic kidney disease (eGFR < 30 mL/min/1.73 m^2^, corresponding to CKD stages 4–5).

Of the final analytical sample (n = 96), six participants (6.25%) had stage 3 CKD (eGFR 30–59 mL/min/1.73 m^2^), while 90 participants (93.75%) had eGFR ≥ 60 mL/min/1.73 m^2^.

During the baseline visit, the primary care physician systematically recorded the main comorbidities and current pharmacological treatments in the case report form. Clinical information was extracted from electronic medical records and verified through structured interview with the participant.

### 2.2. Assessment Procedures

Blood and urine collection: Participants were instructed to fast overnight (minimum 8 h) and attend the clinic in the early morning. Fasting venous blood samples and first-morning spot urine samples were collected. Plasma sodium (Na^+^), chloride (Cl^−^), glucose, and creatinine were measured and estimated glomerular filtration rate (eGFR) was calculated using the CKD-EPI equation (2009). Urinary Na^+^ and Cl^−^ concentrations were determined from spot urine samples.

Bioelectrical impedance analysis (BIA) was performed either in the morning or afternoon depending on participant availability, following a predefined standardization protocol. Participants were instructed to: avoid alcohol, caffeine, and strenuous physical activity for 24 h prior; fast (no food or fluid intake) for at least 4 h before measurement; and void the bladder approximately 30 min beforehand. Body weight and height were measured immediately before BIA using a calibrated Seca 764 scale (Seca GmbH, Hamburg, Germany). Multifrequency BIA was conducted using the InBody S10 device (InBody Co., Seoul, Korea), a segmental analyzer operating at six frequencies (1–1000 kHz) with an eight-point tactile electrode system. Measurements were performed in a temperature-controlled environment (22–24 °C). After removal of all metallic objects, participants rested in the supine position for 10 min to allow fluid redistribution and stabilization. Measurements were performed with the arms abducted and the legs separated at approximately 30° from the body midline. Specifically, variables intended to capture cellular hydration and integrity were included on the basis of bioelectrical parameters [29,30]. Whole-body estimates of phase angle (PhA) at 50 kHz, fat-free mass (FFM), total body water (TBW), and intracellular water (ICW) were recorded.

Muscle strength and functional performance were assessed using standardized procedures. Handgrip strength was measured in the dominant hand using a JAMAR hydraulic dynamometer (Sammons Preston, Bolingbrook, IL, USA), with the handle set at the second position according to standard recommendations. Participants were assessed in a standing position, with the arm fully extended at the side and without external support. Three maximal voluntary contractions were obtained, with a 30 s rest interval between trials to minimize fatigue. The highest value was retained for analysis. Functional performance was evaluated using the Timed Up and Go (TUG) test. Participants were instructed to rise from a standard chair (seat height: 48 cm) without using their arms, walk 3 m at their usual pace, turn, return to the chair, and sit down. Participants were asked to wear comfortable walking footwear, and the use of a habitual assistive device (e.g., cane) was permitted when necessary. Time was recorded in seconds using a digital stopwatch.

### 2.3. Variables for Principal Component Analysis (PCA)

Variables were selected to capture complementary physiological dimensions of water and electrolyte regulation, encompassing cellular hydration, systemic electrolyte homeostasis, and renal electrolyte handling.

Cellular hydration and volume markers (derived from BIA): PhA at 50 kHz, was included as an indicator of cell membrane capacitance and structural integrity, influenced by intracellular water distribution. The ratio of intracellular water to fat-free mass (ICW/FFM) was used to reflect intracellular hydration relative to metabolically active tissue, providing an estimate of cellular water within functional lean mass. The ratio of total body water to body weight (TBW/body weight) represented overall body water as a proportion of total body mass. Although influenced by adiposity, this parameter was included to capture total body water relative to body mass, thereby complementing ICW/FFM, which normalizes intracellular water to lean tissue and is less affected by variations in adiposity.

The ECW/ICW ratio was not included in the PCA model, as simultaneous incorporation of ECW/ICW and ICW/FFM would have introduced mathematical redundancy due to their inherent interdependence and shared variance components, potentially compromising interpretability of the extracted components.

Plasma and urinary electrolyte markers: Na^+^ and Cl^−^ concentrations (mmol/L) were included as indicators of systemic electrolyte homeostasis. Urinary Na^+^ and Cl^−^ concentrations (mmol/L), measured in spot urine samples, were used to reflect renal electrolyte handling. Although spot urine concentrations are influenced by urine dilution, they were analyzed within the multivariate PCA framework alongside plasma electrolytes and cellular hydration markers, allowing the statistical model to identify integrated patterns of hydroelectrolyte regulation.

These seven variables were entered into the principal component analysis to derive composite profiles reflecting multidimensional hydroelectrolytic regulation. Given the sample size (n = 96), the participant-to-variable ratio was approximately 13.7:1, exceeding commonly recommended thresholds (≥5–10 participants per variable) for exploratory PCA. All retained variables showed communalities ≥ 0.40, indicating satisfactory representation within the three-component solution.

Serum glucose, creatinine, and eGFR were measured for participant characterization and to verify exclusion criteria but were not included in the PCA. Fat-free mass index (FFMI = FFM/height^2^) was calculated as an indicator of height-adjusted lean mass and was used as an adjustment variable in subsequent regression analyses examining associations between principal components and muscle function outcomes.

### 2.4. Statistical Analysis

Statistical analyses were performed using XLSTAT version 2023.3. Quantitative variables are presented as mean ± standard deviation, and qualitative variables as absolute frequencies and percentages. Normality was assessed using the Shapiro–Wilk test, and homogeneity of variances using Levene’s test. Comparisons between two groups were performed using Student’s *t* test for independent samples or, when parametric assumptions were not met, the Mann–Whitney U test.

Principal component analysis (PCA) was conducted to explore the underlying multivariate structure of hydration-related variables. PCA was performed using a Spearman correlation matrix in order to reduce sensitivity to non-normal distributions and extreme values, which are common in urinary and electrolyte measurements in older populations.

The analysis was applied as an exploratory multivariate technique aimed at identifying latent physiological dimensions related to hydration status, electrolyte handling and cellular integrity. Principal components were retained based on eigenvalues greater than 1 (Kaiser criterion), inspection of cumulative explained variance, and consistency with physiologically interpretable domains. The primary solution was interpreted using non-rotated components, conceptualized as continuous physiological axes representing integrated hydration domains. As a planned sensitivity analysis, PCA with orthogonal Varimax rotation and Kaiser normalization was additionally performed to assess the stability and interpretability of the component structure.

Component scores derived from the primary PCA solution were subsequently used to characterise and compare hydration profiles. Multivariable linear regression models were constructed to evaluate the associations between principal component scores and both muscle strength (handgrip) and functional performance (TUG). Two sequential regression models were specified: Model 1 adjusted for sex and fat-free mas index (FFMI = FFM/height^2^), to account for baseline differences in lean mass and sex-related variability in muscle function. Model 2 further incorporated age, hypertension, and diuretic use, given their established influence on renal electrolyte handling, fluid distribution, and functional performance.

In all analyses, a *p* value < 0.05 was considered statistically significant.

### 2.5. Ethical Considerations

The study was approved by the Clinical Research Ethics Committee of the Consorci Sanitari del Maresme (CSdM; approval code CEIC CSdM 66/19; date approval: 3 December 2019). All participants provided written informed consent. Data were collected using an electronic case report form and are stored by the CSdM Research Unit in accordance with current data protection legislation (Spanish Data Protection Act and its 2018 update).

## 3. Results

The study population comprised 96 older adults (mean age 75.2 years), with a balanced distribution by sex. Table 1 presents the main anthropometric, clinical and biochemical characteristics of the sample. Men exhibited significantly higher handgrip strength than women (33.6 ± 9.1 kg vs. 18.8 ± 3.9 kg; *p* < 0.01). With regard to functional performance, the mean Timed Up and Go (TUG) time was significantly shorter in men compared with women (9.0 ± 1.9 s vs. 10.7 ± 3.9 s; *p* = 0.044).

Table 2 presents hydration and electrolyte-related parameters stratified by sex, showing that most hydroelectrolytic variables differed significantly between women and men.

Principal component analysis (PCA) identified three components (PC1–PC3) with eigenvalues greater than 1, which together explained 77.5% of the total variance. The first component (PC1) accounted for 36.2% of the variance, followed by the second component (PC2), which explained 20.9%, and the third component (PC3), which explained 20.4% of the total variance.

Factor loadings and the relative contribution of variables in the three retained components are shown in Table 3. The first component (PC1) displayed the highest positive loadings for urinary electrolytes (Na^+^ and Cl^−^), together with relevant contributions from BIA-derived markers related to cellular hydration and volume (ICW/FFM and PhA). Accordingly, PC1 was interpreted as a renal–cellular volume pattern, integrating urinary ion concentration and cellular volume.

The second component (PC2) was dominated by plasma electrolytes, with high positive loadings and minimal contributions from BIA-derived variables and urinary electrolytes. Consequently, PC2 was interpreted as a plasma pattern.

Finally, the third component (PC3) was mainly driven by BIA-derived variables related to cellular hydration and volume, such as ICW/FFM and phase angle (PhA), and was interpreted as a predominantly cellular volume and hydration pattern.

Taken together, these three components reflect complementary patterns of hydroelectrolytic variability, based on different combinations of plasma and urinary electrolytes and BIA-derived indicators, providing an integrative framework for analysing hydroelectrolytic variability in older adults.

Subsequently, principal component scores were analysed in relation to major comorbidities and pharmacological treatments (Table S1). Hypertension was associated with lower scores in PC1 (renal–cellular pattern) and PC2 (plasma electrolyte pattern), whereas no significant differences were observed in PC3 (cellular volume pattern). In contrast, diabetes was associated with lower scores in PC2 and PC3, with no differences in PC1. Diuretic treatment was also related to lower scores in PC1 and PC2, without significant changes in PC3. Finally, blockade of the renin–angiotensin system was associated with lower PC3 scores, and the use of antidiabetic drugs was associated with lower scores in PC2 and PC3, with no differences in PC1.

The Varimax-rotated solution preserved the total explained variance and overall model fit compared with the non-rotated solution, although loadings were redistributed across components. As the non-rotated solution was more consistent with the conceptual framework of integrated physiological domains, it was retained for primary interpretation. Rotated results are provided in the Supplementary Material (Table S2).

To examine the associations between the three principal component scores and handgrip strength (HG) and Timed Up and Go performance (TUG), multivariable linear regression analyses were conducted (Table 4). Two sequential models were fitted. Model 1 included sex and height-adjusted fat-free mass as covariates. Model 2 additionally incorporated age, hypertension, and diuretic treatment to account for major clinical factors related to renal and electrolyte regulation

For handgrip strength, Model 1 explained 61.3% of the variance. In this model, the cellular hydration component (PC3) was significantly associated with strength, whereas the renal–cellular component (PC1) showed a marginal association (*p* = 0.050). In Model 2, which explained 63.0% of the variance after additional clinical adjustment, the association between PC3 remained statistically significant, while PC1 and PC2 were not significantly associated with the outcome.

For TUG performance, Model 1 explained 19.1% of the variance. In this model, both the renal–cellular component (PC1) and the cellular hydration component (PC3) were significantly associated with completion time, whereas the plasma electrolyte component (PC2) was not. In Model 2, the explained variance increased to 34.6%. In this fully adjusted model, PC1 remained significantly associated with TUG performance, PC3 was no longer statistically significant, and PC2 showed a statistically significant association after additional clinical adjustment.

Multivariable linear regression analyses using component scores derived from the Varimax-rotated solution are presented in Supplementary Table S3. The overall explanatory capacity of the models remained comparable to that observed with the non-rotated solution, although the distribution of associations across individual components differed in accordance with the redistribution of loadings after rotation.

## 4. Discussion

The results of this study indicate that muscle strength and functional performance in older adults are associated with integrated patterns of hydroelectrolytic regulation. Using principal component analysis (PCA), an appropriate approach for identifying latent structures in complex biomedical data [31,32], three complementary physiological domains were identified. In Model 1 (adjusted for sex and height-adjusted fat-free mass), the renal–cellular component (PC1) showed a borderline association with handgrip strength, while the cellular hydration component (PC3) demonstrated a modest but statistically significant association. For TUG both PC1 and PC3 were associated with shorter completion time. In Model 2 (additionally adjusted for age, hypertension, and diuretic use), the association between PC3 and handgrip strength remained statistically significant, whereas TUG performance was independently associated with PC1 and, after full adjustment, also with the plasma electrolyte component (PC2). These findings suggest that distinct aspects of muscle function are differentially related to partially overlapping yet physiologically differentiated hydroelectrolytic domains.

From a physiological perspective, these results are consistent with the multicompartmental nature of fluid regulation. The renal–cellular domain (PC1) integrates urinary sodium (Na^+^) and chloride (Cl^−^) concentrations with bioelectrical impedance-derived markers related to intracellular hydration and membrane integrity. This clustering should not be interpreted as a direct causal relationship between electrolyte excretion and cellular volume, but rather as a covariance structure reflecting coordinated regulation between the renal and cellular axes. In classical models of body fluid physiology, membrane ion transport and renal sodium balance are recognized as interdependent yet compartmentally differentiated processes that contribute to maintaining osmotic gradients without necessarily altering plasma electrolyte concentrations [18]. Accordingly, the observed clustering may reflect coordinated regulation across renal and cellular compartments rather than isolated sodium excretion behavior. This domain may capture the mobilization of non-osmotic sodium stores in the interstitium—considered a third compartment with buffering properties—whose dynamics may influence cellular volume in a manner partially uncoupled from plasma natremia [33,34,35].

Although spot urinary Na^+^ and Cl^−^ concentrations are influenced by recent dietary intake and urine dilution, they were not analysed in isolation in the present study. PCA identifies shared variance patterns across multiple physiological variables, enabling the detection of latent domains beyond the behavior of any single marker [31,32]. The consistent loading of urinary electrolytes together with intracellular hydration indices suggests structured covariation that is unlikely to be explained solely by short-term dietary variability.

The plasma electrolyte domain (PC2), characterized by tightly regulated Na^+^ and Cl^−^ concentrations, did not show independent associations with maximal strength. The absence of association with maximal force production may reflect the tight homeostatic regulation of plasma sodium concentration, which primarily represents systemic water balance rather than intracellular fluid distribution [18]. In this sample, plasma electrolyte values displayed limited variability and remained within physiological ranges, which may explain their weaker association with maximal force production. However, in the adjusted model, PC2 was associated with functional performance. This finding suggests that even subtle variations within physiological limits may become relevant when tasks require coordinated systemic responses. TUG performance reflects not only muscle contractile capacity but also a broader physiological reserve integrating cardiovascular, neuromuscular, and volume regulatory components [36,37].

This interpretation is further supported by the increase in explained variance for TUG after clinical adjustment (R^2^ from 0.191 to 0.346), highlighting the contribution of age and comorbidity to functional performance. In contrast, the explanatory capacity for handgrip strength remained stable (R^2^ from 0.613 to 0.630), consistent with a more direct relationship to intracellular muscle properties.

The independent association between the cellular volume domain (PC3) and handgrip strength aligns with studies identifying intracellular water (ICW) as a sensitive marker of muscle quality [38,39]. Maeda et al. [40] demonstrated that intracellular water (ICW) assessment is sensitive to age-related reductions in muscle cell mass that are not fully captured by conventional lean mass estimates derived from DXA or MRI, suggesting that intracellular hydration indices may reflect functional aspects of muscle tissue beyond structural mass alone. Similarly, Germano et al. [41] reported significant associations between phase angle (PhA) and both handgrip strength (adjusted β = 0.024) and gait speed (adjusted β = 0.619), independent of comorbidities and body composition. Morais et al. [42] further demonstrated that PhA independently predicted performance in the 8-foot up-and-go and 6 min walk tests, explaining up to 31.6% of functional variance in older men.

The association between diuretic use and TUG performance showed an inverse direction, with a negative coefficient corresponding to shorter execution times. Several studies linking diuretics to impaired muscle status derives from cohorts with advanced chronic kidney disease or significant cardiovascular comorbidity, whose pathophysiological context differs considerably from that of our population, which consisted predominantly of community-dwelling older adults without clinically significant renal impairment [43,44]. This difference in clinical context may modulate the direction of observed associations. Furthermore, in observational studies, the influence of the healthy user bias cannot be excluded, whereby the use of preventive therapies may act as a marker of structured medical follow-up and a more favorable overall health profile [45,46,47]. Accordingly, this association should be considered exploratory and requires confirmation in longitudinal studies capable of discriminating by pharmacological class and treatment duration.

From a translational perspective, an important implication of these findings is that the variables contributing to the identified components—plasma and urinary Na^+^ and Cl^−^, along with impedance-derived hydration indices—are readily obtainable in primary care settings. Although isolated electrolyte values provide limited functional information, their integration within a multivariate framework may help characterize compartment-specific hydroelectrolytic phenotypes associated with functional capacity in older adults. Longitudinal and interventional studies are needed to determine whether integrated hydroelectrolytic profiling contributes to early identification of functional decline.

Overall, the results suggest a functional hierarchy among hydroelectrolytic domains: the cellular axis appears primarily related to maximal force production; the renal–cellular axis to complex functional integration; and the plasma domain to systemic modulation of performance during tasks requiring multisystem coordination.

These findings support the concept that hydration status in older adults cannot be reduced to a single systemic parameter but rather reflects coordinated interactions among intracellular, renal, and plasma compartments. The differential associations observed for maximal strength and functional performance suggest that distinct functional phenotypes depend on partially overlapping hydroelectrolytic domains. By applying a multivariate framework to capture these integrated physiological axes, the present study highlights the relevance of compartmental fluid regulation in the functional expression of aging skeletal muscle.

This study has several limitations that should be acknowledged. First, its cross-sectional design precludes causal inference, and the observed associations should therefore be interpreted with caution. Although physical activity was not systematically recorded, regression models were adjusted for height-adjusted fat-free mass to partially account for structural differences in muscle mass. In addition, participants had a mean BMI within the overweight range, which may influence fluid distribution and impedance-derived estimates; therefore, extrapolation to populations with substantially different body composition profiles should be approached with caution.

Second, plasma and urinary osmolality were not included. Although osmolality is a central marker of systemic water balance, it is tightly regulated in clinically stable older adults and may have limited sensitivity for detecting compartment-specific alterations, particularly in the presence of preserved renal compensatory mechanisms. For this reason, the analytical focus was directed toward integrated domains of renal handling and cellular hydration.

Finally, although the participant-to-variable ratio met recommended thresholds for exploratory PCA, the relatively modest sample size may limit the stability and generalizability of the extracted components. Replication in larger and longitudinal cohorts will be necessary to confirm these findings.

## 5. Conclusions

In older adults, muscle strength and functional performance are associated with distinct, yet partially overlapping, hydroelectrolytic regulatory domains identified through principal component-derived patterns. The cellular component (PC3) constitutes the primary independent predictor of maximal force production, whereas functional performance (TUG) requires a broader multi-compartmental integration involving renal, plasma, and cellular axes (PC1 and PC2). These findings demonstrate that the functional expression of ageing muscle is intrinsically linked to compartmental fluid homeostasis rather than isolated biochemical levels. Consequently, multivariate analytical frameworks are essential to reveal integrated physiological dimensions of muscle quality that remain obscured when using conventional, isolated biomarkers.

## Figures and Tables

**Table 1:** Characteristics of the study population.

	Total (n = 96)		Women (n = 51)		Men (n = 45)	
Age, years	75.2 ± 4.21		75.39 ± 4.23		74.9 ± 4.11	
BMI	29.15 ± 4.41		29.06 ± 4.99		29.26 ± 3.71	
ASMI, kg/m^2^	7.28 ± 1.08		6.59 ± 0.80		8.06 ± 0.77	
FFM, kg	46.59 ± 8.41		40.64 ± 4.88		53.32 ± 6.18	
FFMI, kg/m^2^	18.26 ± 1.96		17.15 ± 1.57		19.53 ± 1.54	
Comorbidities						
Diabetes	18	(18.75)	8	(15.69)	10	(22.22)
Hypertension	59	(61.46)	33	(64.71)	26	(57.78)
Medications						
Diuretics	37	(38.54)	20	(39.22)	17	(37.78)
Antidiabetics	17	(17.71)	8	(15.69)	9	(20.00)
ACEIs	33	(34.38)	15	(29.41)	18	(40.00)
ARBs	23	(23.96)	12	(23.53)	11	(24.44)
Biochemistry						
Glucose, mg/dL	104.23	±27.09	98.23	±21.04	111.02	±31.50
Creatinine, mg/dL	0.82	±0.19	0.711	±0.13	0.94	±0.17
CKD-EPI, mL/min/1.73 m^2^	79.89	±11.37	80.63	±10.68	79.67	±15.25
Muscle functionality						
HG, kg	25.72	±10.08	18.77	±3.93	33.60	±9.08
TUG, s	9.89	±3.18	10.71	±3.89	9.00	±1.85

Data are presented as mean ± standard deviation (SD) for continuous variables and as number (percentage) for categorical variables. BMI, body mass index; ASMI, appendicular skeletal muscle mass index; FFM, fat-free mass; FFMI, fat-free mass index (FFM/height^2^); ACEIs, angiotensin-converting enzyme inhibitors; ARBs, angiotensin receptor blockers; HG, handgrip strength; TUG, Timed Up and Go test.

**Table 2:** Hydration- and electrolyte-related parameters of the study population by sex.

	Total (n = 96)	Women (n = 51)	Men (n = 45)	*p*
PhA (°)	4.99 ± 0.63	4.70 ± 0.51	5.31 ± 0.59	<0.01
TBW/weight, %	46.64 ± 5.48	44.10 ± 5.16	49.52 ± 4.32	<0.01
ICW/FFM, %	44.98 ± 0.61	44.79 ± 0.61	45.20 ± 0.53	<0.01
Plasma Na^+^, mmol/L	141.55 ± 1.92	141.41 ± 2.00	141.71 ± 1.83	0.593
Plasma Cl^−^, mmol/L	101.18 ± 2.66	101.18 ± 2.83	101.18 ± 2.49	0.767
Urine Na^+^, mmol/L	117.83 ± 47.14	94.93 ± 39.03	143.78 ± 42.06	<0.01
Urine Cl^−^, mmol/L	100.66 ± 46.44	79.63 ± 38.52	124.51 ± 43.3	<0.01

Data are presented as mean ± standard deviation (SD). PhA, phase angle; TBW, total body water; ECW, extracellular water; ICW, intracellular water; FFM, fat-free mass; Na^+^, sodium; Cl^−^, chloride.

**Table 3:** Factor loadings and variance contributions of variables to the principal components (PCA).

	Factor Loadings			Variable Contributions		
	PC1 (Renal/Cellular)	PC2 (Plasma Electrolytic)	PC3 (Cell Volume)	PC1 (Renal/Cellular)	PC2 (Plasma Electrolytic)	PC3 (Cell Volume)
PhA (°)	0.68	−0.23	0.59	18.25	3.74	24.67
TBW/weight, %	0.48	0.13	−0.39	9.02	1.14	11.36
ICW/FFM, %	0.59	−0.07	0.75	13.73	0.32	38.94
Plasma Na^+^, mmol/L	−0.03	0.84	0.18	0.04	48.10	2.16
Plasma Cl^−^, mmol/L	0.13	0.82	0.15	0.65	46.54	1.53
Urine Na^+^, mmol/L	0.88	0.03	−0.36	30.46	0.08	9.19
Urine Cl^−^, mmol/L	0.84	0.03	−0.41	27.84	0.08	11.54

Loadings ≥ 0.40 were considered relevant. The contribution of each variable is expressed as a percentage of the variance of the corresponding component.

**Table 4:** Multivariable associations of principal component scores with handgrip strength and Timed Up and Go performance (Model 1 and Model 2).

	Model 1			Model 2		
Handgrip	β	95% CI	* p *	β	95% CI	* p *
PC1—renal–cellular volume	0.180	(0.000, 0.360)	0.050	0.145	(−0.048, 0.338)	0.138
PC2—plasma electrolyte	−0.089	(−0.222, 0.043)	0.184	−0.109	(−0.250, 0.031)	0.124
PC3—cellular volume	0.152	(0.019, 0.284)	0.025	0.146	(0.010, 0.282)	0.036
Sex	−0.569	(−0.757, −0.381)	<0.001	−0.601	(−0.791, −0.410)	<0.001
FFMI	0.096	(−0.081, 0.272)	0.285	0.063	(−0.119, 0.245)	0.492
Age	—	—	—	−0.121	(−0.258, 0.015)	0.081
Hypertension	—	—	—	0.009	(−0.155, 0.172)	0.917
Diuretics	—	—	—	−0.074	(−0.238, 0.089)	0.369
TUG						
PC1—renal–cellular volume	−0.262	(−0.522, −0.001)	0.049	−0.308	(−0.564, −0.051)	0.019
PC2—plasma electrolyte	−0.128	(−0.319, 0.064)	0.189	−0.190	(−0.376, −0.003)	0.046
PC3—cellular hydration	−0.238	(−0.429, −0.047)	0.015	−0.151	(−0.332, 0.030)	0.102
Sex	0.097	(−0.174, 0.369)	0.478	0.100	(−0.154, 0.353)	0.437
FFM/height^2^	−0.021	(−0.277, 0.234)	0.870	0.042	(−0.200, 0.284)	0.730
Age	—	—	—	0.338	(0.157, 0.520)	<0.001
Hypertension	—	—	—	0.072	(−0.145, 0.289)	0.512
Diuretics	—	—	—	−0.260	(−0.477, −0.042)	0.020

Model 1 adjusted for sex and FFMI. Model 2 additionally adjusted for age, hypertension, and diuretic treatment. Values are standardized regression coefficients (β) with 95% confidence intervals (CI).

## Data Availability

The data presented in this study are available on request from the corresponding author due to ethical and data protection restrictions.

## Supplementary Materials

The following supporting information can be downloaded at: https://www.mdpi.com/article/10.3390/nu18050850/s1, Table S1: Associations between hydroelectrolytic regulation principal components and comorbidities and pharmacotherapy. Table S2: Factor loadings and percentage contributions of variables in the Varimax-rotated PCA solution. Table S3: Sensitivity analysis: multivariable regression models using Varimax-rotated principal component scores.
