# Supplementary material for: Association Between Cellular Hydration Patterns and Hydroelectrolytic Regulation with Muscle Strength in Older Adults
Source: Nutrients. 2026 Mar 5;18(5):850. doi: 10.3390/nu18050850 (PMC12986672; doi:10.3390/nu18050850)
Supplement: Supplementary file 1 [file nutrients-18-00850-s001.zip › nutrients-4159150-supplementary.pdf]

Tabla S1: Associations between hydroelectrolytic regulation principal components and comorbidities and pharmacotherapy

Hypertension	No (n)	No mean (SD)		Yes(n)	Yes mean (SD)		p
F1- renal– volume cellular	37	0.51	(1.60)	59	-0.27	(1.55)	**0.028**
F2- plasma electrolyte		0.45	(1.12)		-0.23	(1.25)	**0.007**
F3- cellular hydration		0.26	(1.31)		-0.14	(1.13)	0.136
Diabetes							
F1- renal– volume cellular	78	-0.07	1.62	18	0.31	1.52	0.405
F2- plasma electrolyte		0.17	1.23		-0.73	0.81	0.003
F3- cellular hydration		0.13	1.22		-0.55	0.95	0.025

Diuretics	No (n)	Without treatment mean (SD)		Yes (n)	With treatment mean (SD)		p
F1- renal– volume cellular	61	0.32	(1.62)	35	-0.56	(1.43)	**0.011**
F2- plasma electrolyte		0.28	(1.14)		-0.48	(1.22)	**0.002**
F3- cellular hydration		-0.15	(1.25)		0.27	(1.08)	0.089
ACEIs							
F1- renal– volume cellular	65	0.11	(1.63)	31	-0.23	(1.54)	0.348
F2- plasma electrolyte		0.06	(1.16)		-0.13	(1.34)	0.449
F3- cellular hydration		0.14	(1.13)		-0.29	(1.30)	0.148
ARBs							
F1- renal– volume cellular	73	0.08	(1.63)	23	-0.25	(1.52)	0.337
F2- plasma electrolyte		0.08	(1.12)		-0.24	(1.48)	**0.261**
F3- cellular hydration		-0.19	(1.16)		0.59	(1.15)	**0.005**
Antidiabetics							
F1- renal– volume cellular	79	-0.03	(1.95)	17	0.15	(1.41)	0.668
F2- plasma electrolyte		0.15	(1.24)		-0.70	(0.83)	**0.004**
F3- cellular hydration		0.13	(1.21)		-0.62	(0.94)	**0.016**

Principal component scores according to comorbidities and pharmacotherapy. Data are presented as mean (SD). Comparisons were performed between participants with and without each comorbidity or treatment using independent-samples tests, as appropriate. *p* values indicate between-group differences in component scores. ACEIs, angiotensin-converting enzyme inhibitors; ARBs, angiotensin receptor blockers.

Table S2: Factor loadings and percentage contributions of variables in the Varimax-rotated PCA solution.

	Factor loadings			Variable contributions		
	PC1R	PC2R	PC3R	PC1R	PC2R	PC3R
PhA (°)	0.18	-0.09	0.91	1.51	0.54	45.68
TBW/weight, %	0.64	0.06	-0.07	19.01	0.25	0.24
ICW/FFM, %	0.03	0.10	0.95	0.05	0.69	49.44
Plasma Na^+, mmol/L	-0.05	0.86	-0.03	0.12	50.03	0.05
Plasma Cl^-, mmol/L	0.09	0.84	0.04	0.40	48.45	0.11
Urine Na^+, mmol/L	0.92	-0.01	0.23	39.69	0.01	2.87
Urine Cl^-, mmol/L	0.92	-0.02	0.17	39.21	0.03	1.61

Loadings ≥ 0.40 were considered relevant. PCA was performed using Varimax rotation with Kaiser normalization. Variable contributions are expressed as percentages of the variance explained by each rotated component.

Table S3: Sensitivity analysis: multivariable regression models using Varimax-rotated principal component scores.

Handgrip	β	CI	*p*
PCR1	0.022	(-0.160, 0.203)	0.813
PCR2	-0.073	(-0.211, 0.064)	0.293
PCR3	0.220	(0.067, 0.374)	0.005
Sex	-0.601	(-0.791, -0.410)	**<0.001**
FFMI	0.063	(-0.119, 0.245)	0.492
Age	-0.121	(-0.258, 0.015)	0.081
Hypertension	0.009	(-0.155, 0.172)	0.917
Diuretics	-0.074	(-0.238, 0.089)	0.369
**TUG**			
PCR1	-0.177	(-0.419, 0.065)	0.149
PCR2	-0.226	(-0.409, -0.043)	0.016
PCR3	-0.267	(-0.471, -0.063)	0.011
Sex	0.100	(-0.154, 0.353)	**0.437**
FFMI	0.042	(-0.200, 0.284)	0.73
Age	0.338	(0.157, 0.520)	<0.001
Hypertension	0.072	(-0.145, 0.289)	0.512
Diuretics	-0.260	(-0.477, -0.042)	0.02

β coefficients are standardized regression coefficients.
